# Cellular Immune Responses to Nine *Mycobacterium tuberculosis* Vaccine Candidates following Intranasal Vaccination

**DOI:** 10.1371/journal.pone.0022718

**Published:** 2011-07-25

**Authors:** Suraj B. Sable, Mani Cheruvu, Subhadra Nandakumar, Sunita Sharma, Kakali Bandyopadhyay, Kathryn L. Kellar, James E. Posey, Bonnie B. Plikaytis, Rama Rao Amara, Thomas M. Shinnick

**Affiliations:** 1 Division of TB Elimination, National Center for HIV/AIDS, Viral Hepatitis, STD, and TB Prevention, Centers for Disease Control and Prevention, Atlanta, Georgia, United States of America; 2 Department of Microbiology and Immunology, Emory Vaccine Center and Yerkes National Primate Research Center, Emory University, Atlanta, Georgia, United States of America; 3 Biotechnology Core Facility Branch, Division of Scientific Resources, Centers for Disease Control and Prevention, Atlanta, Georgia, United States of America; French National Centre for Scientific Research - Université de Toulouse, France

## Abstract

**Background:**

The identification of *Mycobacterium tuberculosis* vaccines that elicit a protective immune response in the lungs is important for the development of an effective vaccine against tuberculosis.

**Methods and Principal Findings:**

In this study, a comparison of intranasal (i.n.) and subcutaneous (s.c.) vaccination with the BCG vaccine demonstrated that a single moderate dose delivered intranasally induced a stronger and sustained *M. tuberculosis*-specific T-cell response in lung parenchyma and cervical lymph nodes of BALB/c mice than vaccine delivered subcutaneously. Both BCG and a multicomponent subunit vaccine composed of nine *M. tuberculosis* recombinant proteins induced strong antigen-specific T-cell responses in various local and peripheral immune compartments. Among the nine recombinant proteins evaluated, the alanine proline rich antigen (Apa, Rv1860) was highly antigenic following i.n. BCG and immunogenic after vaccination with a combination of the nine recombinant antigens. The Apa-induced responses included induction of both type 1 and type 2 cytokines in the lungs as evaluated by ELISPOT and a multiplexed microsphere-based cytokine immunoassay. Of importance, i.n. subunit vaccination with Apa imparted significant protection in the lungs and spleen of mice against *M. tuberculosis* challenge. Despite observed differences in the frequencies and location of specific cytokine secreting T cells both BCG vaccination routes afforded comparable levels of protection in our study.

**Conclusion and Significance:**

Overall, our findings support consideration and further evaluation of an intranasally targeted Apa-based vaccine to prevent tuberculosis.

## Introduction

*Mycobacterium tuberculosis*, the causative agent of tuberculosis (TB), continues to ravage mankind throughout the world. According to the World Health Organization (WHO) an estimated 1.7 million deaths occurred in 2009 due to TB. Despite a prediction of decline in global incidence, the number of new TB cases continues to grow, approaching 10 million in 2010 [1]. The live attenuated *Mycobacterium bovis* bacillus Calmette-Guerin (BCG) vaccine is the only TB vaccine currently licensed for human use. The vaccine is recommended by the WHO and is administered intradermally as a part of childhood immunization programs in many countries. Although the BCG vaccine is considered to be effective against severe pediatric and extra-pulmonary forms of TB, the vaccine has failed to confer effective protection against adult pulmonary TB in developing countries. Several clinical and field trials have demonstrated that the protective efficacy of the vaccine is highly variable. Development of improved prophylactic and therapeutic interventions has been emphasized to control the TB pandemic [2], [3], [4].

Currently, many new TB vaccine candidates are being evaluated in preclinical studies, and a few have progressed to human clinical trials, such as recombinant BCG (r-BCG) and subunit vaccines [Stop TB Partnership Working Group on New TB Vaccines, Vaccine Pipeline, 2009. http://www.stoptb.org/wg/new_vaccines]. The potential uses of the vaccines vary in different scenarios depending on the age, immunocompetence, BCG vaccination history and exposure to *M. tuberculosis* or environmental mycobacteria of the vaccinee. The new vaccines may be used as a pre-exposure priming or a post-exposure booster vaccine to prevent disease or even as a therapeutic vaccine for individuals with active TB [5]. Each of these strategies is aimed at preventing or eliminating the disease rather than preventing infection.

Because TB is primarily a respiratory disease, it has been hypothesized that vaccination directed at the respiratory mucosa may provide the best opportunity for protection against infection with the tubercle bacillus. Recent studies have investigated intranasal (i.n.) vaccination as a means to stimulate mucosal immunity to TB (reviewed in [6], [7]). Intranasal vaccination offers a needle-free means of a safe and effective immunization against many mucosal pathogens [8] and has numerous advantages over the oral route of vaccination [6]. Intranasal instillation in the nostril also provides a relatively safer strategy compared to deep lung delivery using inhalation or aerosol vaccination [9]. Because responses induced by i.n. vaccination are not influenced by preformed systemic immunity, it also offers an important advantage over parenteral vaccination, which may be less effective in individuals who have pre-existing antibodies [10]. This is of particular importance for vaccination strategies against TB in mothers and infants in developing countries, where prior Th2 background immunity due to extensive exposure to helminthes and saprophytic mycobacteria has been hypothesized to sabotage protective anti-mycobacterial Th1 imprinting and led to BCG failure [11].

It is envisaged that i.n. vaccination that targets the site of entry of *M. tuberculosis* bacilli (i.e., lung) would be able to prevent infection and subsequent TB disease in the host [12]. Previous studies in animal models have shown that i.n. delivery of live or killed BCG vaccine [7], [13], [14], protein subunit vaccines [7], [15], [16], [17], lipoglycan-protein conjugate vaccine [18], plasmid DNA [19], messenger RNA [20], recombinant bacterial vector [21], [22] or viral vector vaccines expressing *M. tuberculosis* proteins [23], [24] induce a stronger immune response or imparts improved protection against *M. tuberculosis* challenge than subcutaneous (s.c.) or other parenteral routes of vaccination [7], [14], [23], [24]. Both lung resident and airway luminal T cells have been suggested to play an important part in i.n. vaccine induced protection against *M. tuberculosis* challenge in the mouse model [25], [26]. However, the mechanism of improved protection following i.n. vaccination is not fully understood and the immune responses generated at different mucosal and systemic immune compartments by live BCG or subunit i.n. vaccinations have not been studied in detail.

In this study, we evaluated mycobacterium-specific immune responses generated at different local and systemic immune sites following a single, quantitatively moderate but effective protective dose of live BCG vaccine administered via the i.n. or s.c. route in a BALB/c mouse model. Our results demonstrate the capacity of i.n. BCG vaccination to induce strong T-cell responses in the lung parenchyma and cervical lymph nodes (CLN), and to impart protection against airway *M. tuberculosis* challenge. We also evaluated nine putative *M. tuberculosis* vaccine candidates for their recognition by immune cells of the lungs and other local or systemic compartments following i.n. BCG or multicomponent-subunit vaccination in order to identify relevant component(s) for a future mucosal TB vaccine.

## Results

### Intranasal BCG vaccination induces significantly stronger type 1 response in the lungs than subcutaneous vaccination

A moderate dose of BCG (5×10^5^ CFUs) has previously been shown to be protective when administered by the i.n. route [14]. This dose (∼0.5–1×10^6^ CFUs) is also recommended for preclinical vaccine testing in the mouse model by the s.c. route against virulent *M. tuberculosis* challenge (Brennan M. J., personal communication; when at Food and Drug Administration, Bethesda, MD, USA). After i.n. or s.c. vaccination with a moderate dose of BCG, the frequency and distribution of IFN-γ, IL-2, and IL-4 secreting antigen-specific cells in the lungs, spleen and the respective draining lymph nodes of BALB/c (H-2^d^) mice were measured over a course of 12 weeks by ELISPOT assay.

The total number of cells isolated from the different organs did not significantly differ between diluent-administered control mice and mice given BCG by either route except for respective draining lymph nodes. Approximately three-fold more cells were obtained from the cervical lymph nodes (CLN) of i.n. BCG vaccinated mice than those of s.c. BCG or diluent-administered mice, while three-fold to four-fold more cells were isolated from the inguinal lymph nodes (ILN) of s.c. BCG vaccinated mice than those of i.n. BCG or diluent-administered mice at 12 weeks. The frequency of cytokine secreting cells observed in *in vitro* cultures after stimulation with tetanus toxoid (TT) compared to those unstimulated was not statistically different in cell number adjusted ELISPOT assays for all organs evaluated, indicating that the observed increased responses after *M. tuberculosis* whole cell lysate (WCL) stimulation were mycobacterium specific (data not shown). WCL-specific cytokine responses of lung and spleen cell cultures were found to be abrogated by CD4^+^ T-cell depletion, but were least affected by depletion of CD8^+^, γδ or NK1.1 T-cells as evaluated at 6 weeks after i.n. BCG vaccination (data not shown).

**Figure 1:**
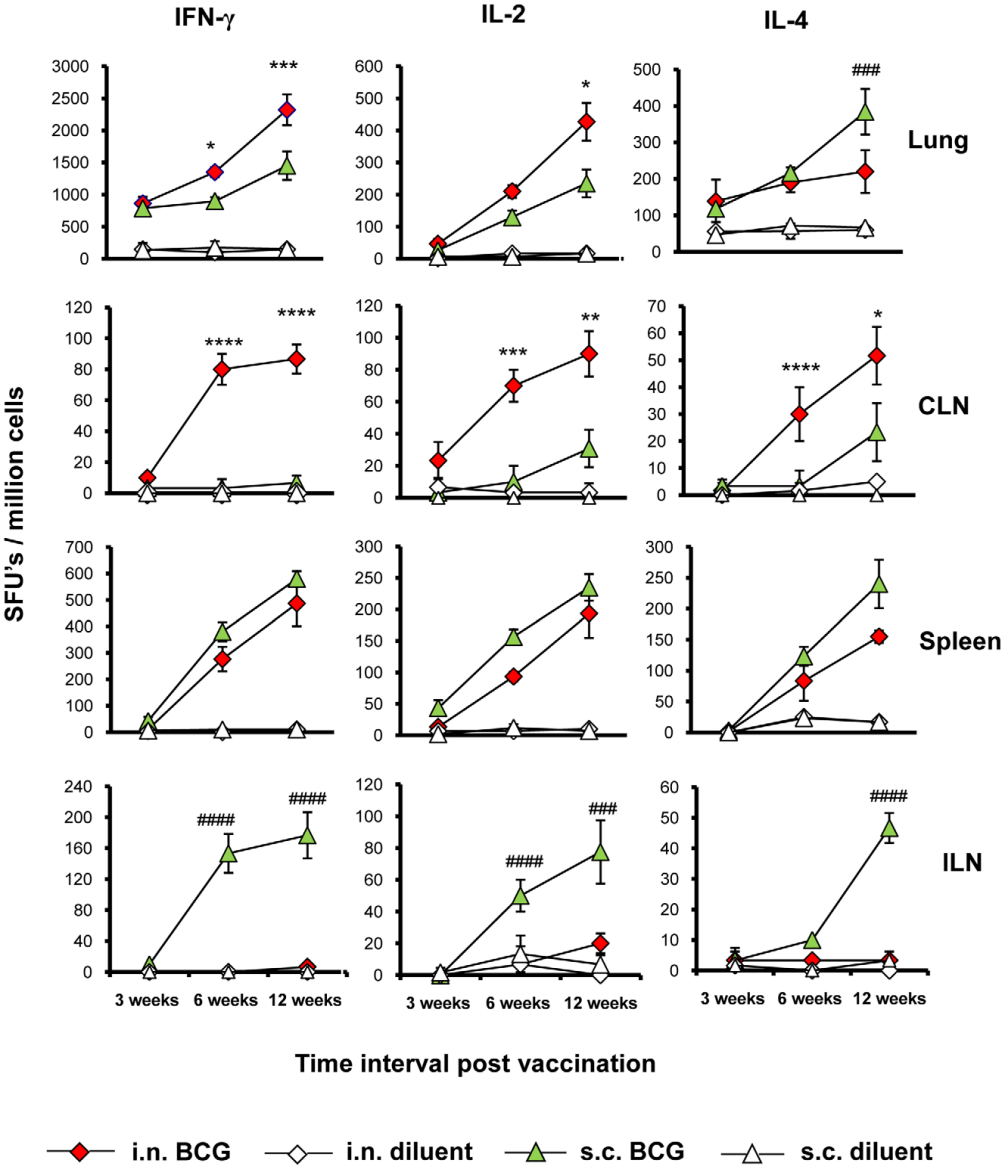
Kinetics of T-cell responses induced by intranasal or subcutaneous BCG vaccination. The frequencies of *M. tuberculosis* whole cell lysate (WCL) specific IFN-γ, IL-2 and IL-4 cytokine secreting cells were enumerated in lungs, cervical lymph nodes (CLN), spleen and inguinal lymph nodes (ILN) of BALB/c mice at 3, 6 and 12 weeks post BCG or diluent (control) vaccination by ELISPOT assay. The frequencies are expressed as spot forming units (SFUs)/million cells of organ after *in vitro* stimulation of 1×10^5^ cells/well from each organ with WCL for 36–40 h in the presence of BM-DCs at the ratio of 5∶1 organ cells/DC. The results are calculated as means ± standard deviation of three to six determinations of pooled cells from four mice after subtracting the SFUs from respective unstimulated cultures. Data presented are representative of three similar experiments. Increased frequencies of WCL-specific IFN-γ secreting cells in the lungs of intranasal BCG vaccinated mice were confirmed by evaluation of individual mice responses at 12 weeks in one of the three experiments. Significant differences among BCG vaccinated groups determined by ANOVA are shown. *: p<0.05; **: p<0.01; ***: p<0.001 ****: p<0.0001 *versus* s.c. BCG and #: p<0.05; ##: p<0.01; ###: p<0.001 ####: p<0.0001 *versus* i.n. BCG.

Vaccination by i.n. route induced more WCL-specific IFN-γ, IL-2 and IL-4 secreting cells in the lung and draining CLN as compared to the s.c. route at all three time points evaluated (Fig. 1), except at the 12 week time point where IL-4-secreting spot forming units (SFU's) in the lungs were higher following s.c. BCG vaccination (p<0.001). Conversely, s.c. BCG vaccination induced higher WCL-specific IFN-γ, IL-2 and IL-4 responses in the ILN, which drains the flank (the site of s.c. vaccination), and spleen as compared to i.n. vaccination at all time points evaluated. However, the splenic cytokine responses of the s.c. group were not statistically different than those of the i.n. group (p>0.05), which indicates that i.n. and s.c. BCG vaccination induce comparable levels of WCL-specific T-cell responses in the spleen. Overall, while both i.n. and s.c. BCG vaccination produce strong splenic T-cell responses, i.n. BCG elicits significantly higher type 1 (IFN-γ, IL-2) responses in the lungs.

### Intranasal BCG vaccination induces significantly higher levels of nitric oxide (NO) in the lungs than subcutaneous vaccination

**Figure 2:**
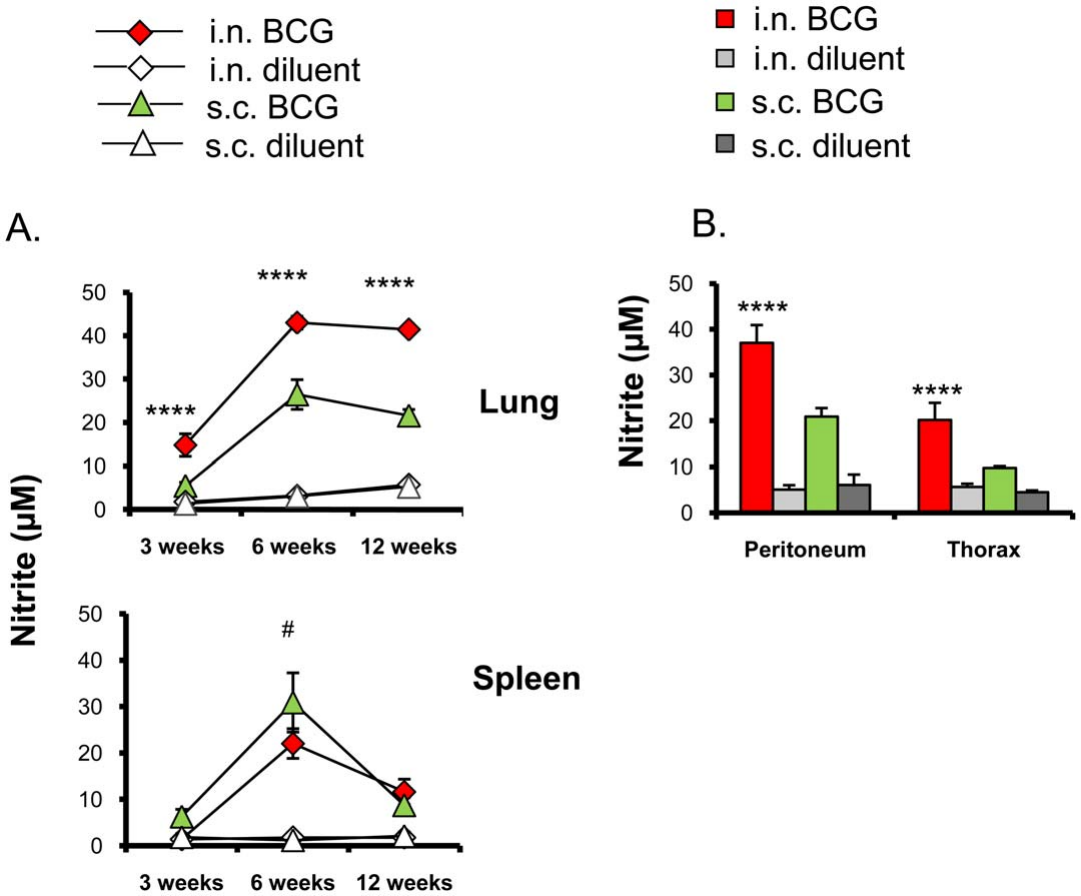
Kinetics of NO response induced by intranasal or subcutaneous BCG vaccination. **A.** The levels of nitrite released after *in vitro* stimulation with *M. tuberculosis* whole cell lysate (WCL) were estimated in lung and spleen cell culture supernatants at 3, 6 and 12 weeks post vaccination of BALB/c mice by Griess assay. The levels are expressed as µM nitrite/million cells of the organ after 96 h of stimulation with WCL. **B.** Nitrite levels produced by WCL-stimulated exudate cells isolated from peritoneal cavity (PC) and thoracic cavity (TC) at 12 weeks. The results are presented as means ± standard deviation of three WCL-stimulated independent culture supernatants assayed in duplicate after subtracting the background nitrite levels from respective unstimulated cultures. The nitrite levels of unstimulated cultures were always less than 5 µM. Significant differences determined by ANOVA are shown. ****: p<0.0001 *versus* s.c. BCG and #: p<0.05 *versus* i.n. BCG.

We next evaluated the innate immune response in terms of NO production induced by either i.n. or s.c. BCG vaccination. Lung cell cultures of i.n. BCG vaccinated mice produced significantly higher nitrite levels following WCL stimulation at all the three time points evaluated than those of s.c. BCG (p<0.0001) or diluent-administered mice (p<0.0001) (Fig. 2A). Conversely, spleen cell cultures of s.c. BCG vaccinated mice produced significantly higher nitrite levels (p<0.05) than those of i.n. BCG vaccinated mice at the 6 week time point (Fig. 2A).

Furthermore, thoracic and peritoneal exudate cells following i.n. BCG vaccination were found to produce significantly higher levels of nitrite after stimulation with WCL (p<0.0001) than those isolated following s.c. BCG vaccination at 12 weeks (Fig. 2B). These results suggest the ability of i.n. BCG to induce strong activation of lung and thoracic innate immune cells such as macrophages and dendritic cells to produce more NO.

### Intranasal BCG vaccination induces long-lasting T-cell responses in different immune compartments

**Figure 3:**
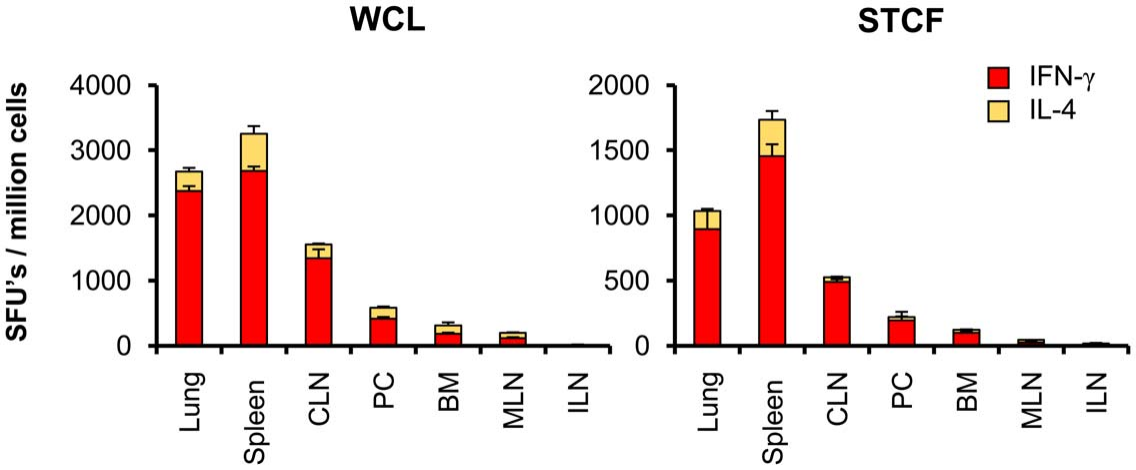
The distribution of T-cell responses in different immune sites after intranasal BCG vaccination. The distribution of *M. tuberculosis* whole cell lysate (WCL) and short term culture filtrate (STCF) specific T-cells in local and peripheral immune compartments of BALB/c mice was investigated at 30 weeks after intranasal BCG vaccination. The frequencies of IFN-γ and IL-4 secreting cells in lungs, spleen, cervical lymph nodes (CLN), peritoneal cavity (PC), bone marrow (BM), mesenteric lymph nodes (MLN), and inguinal lymph nodes (ILN) were enumerated by ELISPOT assay. The frequencies are expressed as spot forming units (SFUs)/million cells after *in vitro* stimulation of 1×10^5^ cells/well from each organ for 36–40 h in the presence of BM-DCs at the ratio of 5∶1 organ cells/DC. The results are calculated as means ± standard deviation of three to six determinations of pooled cells from four mice after subtracting the SFUs from respective unstimulated cultures.

The ability of i.n. BCG vaccination to induce mycobacterium-specific IFN-γ, IL-2 and IL-4 cytokine responses in various local and distant immune compartments was evaluated by ELISPOT and lymphocyte proliferation assay at 6 and 30 weeks after vaccination using two *M. tuberculosis* antigen preparations, short term culture filtrate (STCF) and WCL, as *in vitro* stimulants. STCF represents a mixture of secreted antigens whereas WCL contains antigens of subcellular origin. Intranasal BCG vaccination induced both STCF- and WCL-specific long-term T-cell responses in the lungs, the local lymph nodes draining the nasal passage (i.e., CLN) and the spleen as evaluated by ELISPOT assay at 30 weeks post-vaccination. In these organs T-cell response was characterized by increased frequencies of IFN-γ compared to IL-4 secreting SFU's (Fig. 3). The specific cytokine responses were also observed in the peritoneal cavity (PC), bone marrow (BM) and mesenteric lymph nodes (MLN) but not in the ILN at 30 week time point (Fig. 3). Increased frequencies of cytokine secreting SFU's were observed at late (30 weeks) compared to early (6 weeks) time point in all these organs, however, the WCL-specific IFN-γ responses in the MLN declined by 2.3-fold and IL-4 responses in the BM declined by 2.8-fold at 30 weeks (data not shown). Although cells obtained from the nasal associated lymphoid tissue (NALT) were not evaluated in ELISPOT assays, NALT demonstrated comparable levels of STCF- and WCL-specific proliferation at early (mean stimulation index (SI) 12.15 and 18.60 at 6 weeks respectively; mean CPM of unstimulated culture 720) and late (mean SI 10.26 and 12.52 at 30 weeks respectively; mean CPM of unstimulated culture 660) time points (data not shown). The cells isolated from different immune compartments of naïve and diluent-administered mice, however, did not induce any antigen-specific response.

### Alanine-proline-rich antigen (Apa, Rv1860) induces strong IFN-γ response following intranasal BCG vaccination

**Figure 4:**
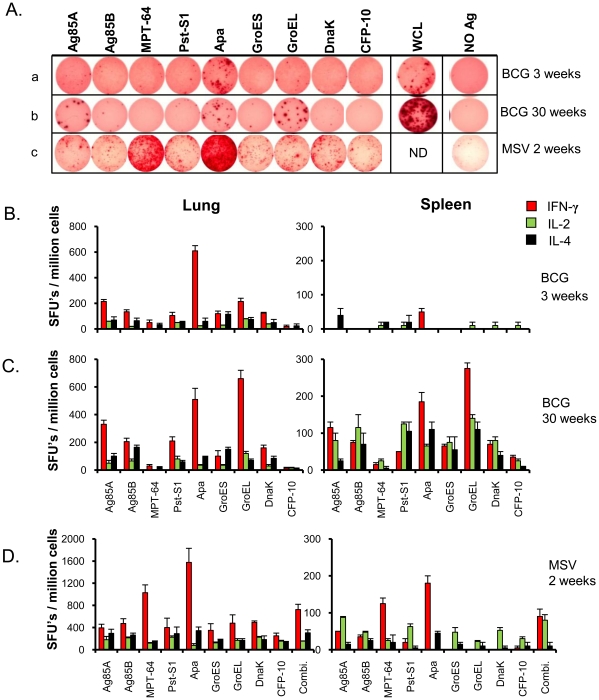
T-cell responses induced by *M. tuberculosis* antigens in intranasally BCG or multicomponent subunit-vaccinated mice. **A.** Representative of nine different *M. tuberculosis* recombinant antigens-specific IFN-γ spot forming units (SFUs) elicited in the lungs *in vitro* at 3 weeks (panel a) and 30 weeks (panel b) after BCG vaccination and 2 weeks after last dose of multicomponent subunit vaccination (panel c) of BALB/c mice. Multicomponent subunit vaccine (MSV) was comprised of a cocktail of nine different proteins in DDA-MPL adjuvant. The IFN-γ ELISPOT assay was developed after stimulation of 1×10^5^ or 0.5×10^5^ pooled lung cells from four vaccinated mice/well for 40 h with individual antigens or WCL in the presence of BM-DCs at the ratio of 5∶1 lung cells/DC. The WCL was not evaluated (ND) in case of MSV. (**B**, **C** and **D**) The comparative frequencies of nine antigen-specific IFN-γ, IL-2 and IL-4 cytokine secreting cells in lungs and spleen of mice at **B.** 3 weeks and **C.** 30 weeks after BCG or **D.** 2 weeks after multicomponent subunit vaccination. The assay was performed using 10 µg/ml of individual antigen or total combination (Combi.) for *in vitro* stimulation in the presence of BM-DCs. The results are presented as SFUs/million cells of organ and are calculated as means ± standard deviation of two to four determinations of pooled cells from four mice after subtracting the SFUs from respective unstimulated cultures. The data presented are representative of two similar experiments.

We compared nine recombinant *M. tuberculosis* vaccine candidates (Table 1) [27], [28], [29], [30], [31], [32], [33], [34], [35], [36], [37], [38], [39] for their ability to be recognized by T cells at 3 and 30 weeks following i.n. vaccination with BCG. These candidates are known to induce strong systemic immunity in humans and/or experimental animals when administered in different vaccine forms by parenteral route (i.e. percutaneous or intramascular injection), and impart significant protection against mycobacterial challenge in different animal models in preclinical studies (Table 1). Fig. 4 shows the frequency of IFN-γ, IL-2 and IL-4 cytokine-secreting cells in lung and spleen following *in vitro* stimulation with individual candidates. Among all antigens evaluated, Apa induced the strongest IFN-γ response at the early time point while GroEL, Apa and Ag85A were the top three inducers of IFN-γ response at the later time point in the lungs (Fig. 4A; panel a, b). Recognition of all antigens was higher in the lungs at the 3-week time point than in cells from the spleen (Fig. 4B). The Apa-induced IL-2 response was low as compared to the GroEL-induced response at both time points evaluated. The frequency of Apa-specific IL-4 producing cells was also less as compared to IFN-γ secreting cells in the lungs and spleen at both 3 and 30 weeks (Fig. 4B, C). Consistent with the observations in the lungs, higher frequencies of Apa-specific IFN-γ producing cells were observed in the CLN at 3 weeks while GroEL, Apa and Ag85A induced a stronger IFN-γ response at 30 weeks (Fig. S1). As expected, cytokine responses induced by CFP-10 and MPT-64 were either totally absent or very low since these antigens are absent from the BCG Copenhagen strain used for vaccination (Fig. 4B, C and S1).

**Table 1:** Recombinant *M. tuberculosis* proteins evaluated in the study.

Vaccine candidate^a^	Sanger annotation	Calculated molecular wt (kDa)^b^	Observed molecular wt (kDa)^c^	Lot number	Endotoxin Level (ng/mg)^d^	Function	Ref
Ag85A	Rv3804c	35.55	30.5	05.rEC.04.28.Ag85A	1.37	Mycolyl Transferase/Fibronectin binding	[27], [28]
Ag85B	Rv1886c	34.45	29.5	05.rEC.04.25.Ag85B	1.43	Mycolyl Transferase/Fibronectin binding	[29], [30], [31]
MPT-64	Rv1980c	24.72	24.0	05.rEC.05.25.MPT64	1.23	Unknown	[32], [33]
Pst-S1	Rv0934	38.11	38.0	05.rEC.07.12.Psts1	1.90	Phosphate transporter	[34]
Apa	Rv1860	32.59	45–47	05.rEC.06.03.ModD	1.77	Fibronectin binding/host cell attachment	[35]
GroES	Rv3418c	10.67	10.0–12.0	02.rEc.06.12.GroES	0.69	Chaperonin(HSP10)/ATPase	[36]
GroEL	Rv0440	56.70	65.0	05.rEC.06.08.GroEL2	2.09	Chaperonin (HSP65)	[37]
DnaK	Rv0350	66.70	70.0	03.rEC.07.21.DnaK	0.86	Chaperonin(HSP70)/ATPase	[38]
CFP-10	Rv3874	10.66	10.0	05.rEC.07.12.CFP10	1.67	Unknown/ESAT-6 family member	[39]

a Obtained through the NIH-TB vaccine testing and research material contract.

b Obtained from http://www.ncbi.nlm.nih.gov/entrez.

c Molecular weight of native protein observed in SDS-PAGE analysis.

d Endotoxin levels were determined by Limulus amebocyte lysate (LAL) assay.

### Apa induces strong IFN-γ response following intranasal multicomponent subunit vaccination

The ability of individual vaccine candidates to induce T-cell responses following multicomponent subunit vaccination was evaluated. Fig. 4 shows the frequency of IFN-γ, IL-2 and IL-4 cytokine secreting cells in lung and spleen after *in vitro* stimulation with individual candidates. Following i.n. cocktail vaccination in Dimethyl-dioctadecyl-ammonium bromide (DDA)-monophosphoryl lipid A (MPL) adjuvants, all nine vaccine candidates were found to be strongly recognized by T-lymphocytes from the lungs as evaluated by ELISPOT assay. The top two candidates in terms of induction of IFN-γ secreting cells at the level of the lungs were Apa and MPT-64 (Fig. 4A; panel c). The antigen recognition pattern was similar in all the immune compartments evaluated with the exception of MLN (Fig. 4D and S2). Although Apa induced both IFN-γ and IL-4 producing cells at the majority of sites following vaccination, the frequency of IL-2 secreting cells was low. On the other hand, Ag85 complex (A and B), Pst-S1 and DnaK were observed to be prominent inducers of IL-2 secreting cells among all candidates evaluated (Fig. 4D and S2). Furthermore, all nine proteins induced strong proliferation of lung cells after *in vitro* stimulation as evaluated by ^3^H thymidine uptake assay after 72 h of culture. The top three candidates inducing lung cell proliferation were Pst-S1, Ag85A and Ag85B, similar to immunogen-specific IL-2 response (data not shown). None of the immunogens were mitogenic in sham (DDA-MPL) immunized mice, and their ELISPOT cytokine responses were either equal to respective unstimulated cultures from vaccinated mice or below detection limits (data not shown).

The results of multiplexed microsphere-based cytokine immunoassays to measure cytokine induction by individual proteins demonstrated that elevated levels of IL-12(p70), TNF-α, GM-CSF, IL-4, and IL-10 were secreted in lung cell culture supernatants in response to Apa stimulation after 72 h of *in vitro* culture. Among the purified antigens evaluated, Ag85A, Ag85B and Pst-S1 induced the strongest IL-2 response while IL-2 levels were lowest in the Apa stimulated culture supernatants (Table 2).

**Table 2:** Th1 and Th2 cytokines induced by intranasal multicomponent subunit vaccination.

Cytokine Released (pg/ml)*	Protein(s) used for *in vitro* stimulation												
	No Ag	Ag85A	Ag85B	MPT-64	Pst-S1	Apa	GroES	GroEL	DnaK	CFP-10	Cocktail	STCF	WCL
IL-2	18.4	859.3	863.4	424.1	739.9	176.6	525.4	393.5	659.9	367.6	700.0	609.0	778.0
IL-12 (p70)	BDL	4.2	8.3	24.0	1.0	24.0	BDL	8.3	4.2	BDL	16.2	20.1	31.7
TNF-α	4.1	23.2	32.7	47.0	32.7	47.5	27.9	42.2	23.2	13.7	56.5	58.9	258.6
GM-CSF	75.0	130.1	161.1	162.8	111.9	203.9	136.4	106.9	157.5	96.3	594.7	467.3	753.6
IL-4	2.9	33.3	24.9	42.1	20.3	67.9	13.8	16.2	14.7	15.7	54.0	35.0	44.0
IL-5	193.7	267.1	352.9	266.7	118.6	203.6	212.8	148.4	382.1	209.1	745.4	579.7	876.5
IL-10	43.9	51.3	65.8	55.0	43.9	102.9	51.3	51.3	62.2	43.9	131.5	156.0	399.1

*The results are expressed as mean pg/ml levels in *in vitro* antigen stimulated duplicate 1×10^6^ lung cells/ml of RPMI culture as estimated by multiplexed cytokine bead-based assay. The standard deviation between replicate wells was less than 10%.

BDL; below detection limit.

### Intranasal Apa vaccine imparts significant protection in a mouse TB infection model

**Figure 5:**
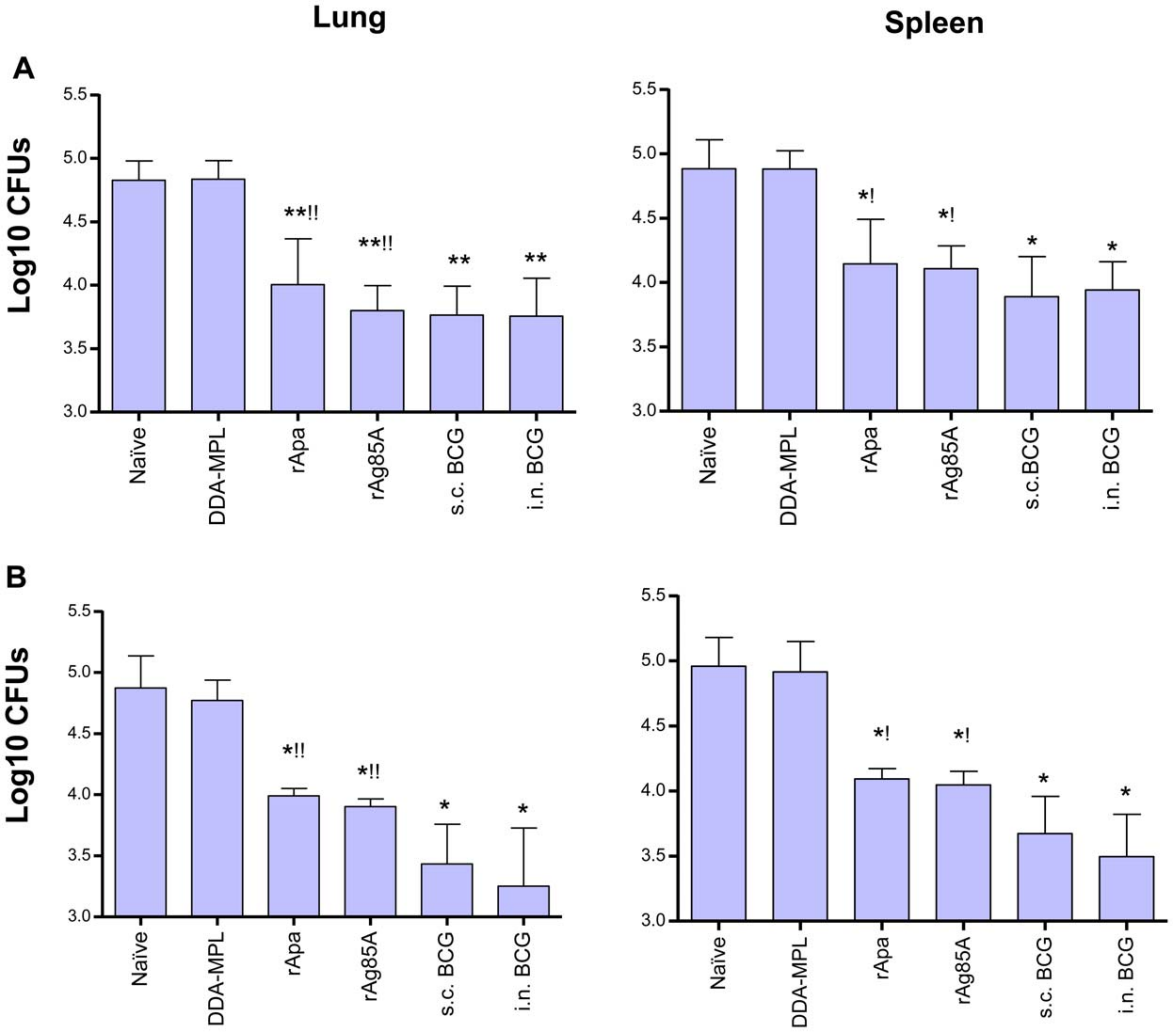
Protective efficacy of intranasal Apa/DDA-MPL subunit vaccine. In two independent experiments (**A** and **B**) groups of BALB/c mice were vaccinated intranasally with three different doses of *M. tuberculosis* recombinant Apa or Ag85A in DDA-MPL and compared to unvaccinated naïve, adjuvant alone and BCG vaccinated controls. In experiment-A three subunit vaccine doses were instilled at two-week intervals while in experiment-B they were administered at four-week intervals. All groups were challenged by the intranasal route with virulent *M. tuberculosis* Erdman either eight weeks (experiment A) or twelve weeks (experiment B) after the first vaccination. Six weeks post-challenge, all mice were sacrificed and the bacterial burden (CFU's) was measured in the lungs and spleen. In both experiments data are presented as mean values from five mice per group and standard deviation of the means are indicated by error bars. Statistical comparisons among the groups were done by one-way ANOVA and Tukey's test. Significant differences are shown. **: p<0.01, *: p<0.05 with respect to (wrt) naïve controls, and ^!!^: p<0.01, ^!^: p<0.05 wrt DDA-MPL adjuvant controls. The significant differences (p<0.05) between the multiple groups were also confirmed by nonparametric Kruskal-Wallis test.

Having observed that Apa is antigenic with i.n. BCG and strongly immunogenic in multicomponent subunit vaccinated mice, we examined the protective efficacy of i.n. Apa/DDA-MPL subunit vaccine using two different vaccination schedules. In one experiment, mice were vaccinated with Apa/DDA-MPL vaccine three times at two-week intervals while in another experiment three doses were administered at four-week intervals. As intranasal Ag85A vaccination has been previously shown to be protective in the mouse model [7], [23], [24], [40], i.n. Ag85A/DDA-MPL subunit vaccinated mice were included as a positive control for protection along with i.n. and s.c. BCG vaccinated mice. Four weeks after the last subunit vaccination, the mice were challenged by intranasal route with virulent *M. tuberculosis* and bacterial numbers were assessed in the lungs and spleen six weeks post challenge. Consistent with antigenicity and immunogenicity studies, Apa subunit vaccination imparted significant protection in terms of reduction of bacterial numbers in the target organs examined (Fig. 5, which shows the results of two different vaccination experiments). The CFUs were significantly lower in the lungs and spleen of Apa vaccinated mice as compared to both unvaccinated naïve and adjuvant alone immunized control groups (ANOVA; p<0.05). The level of protection imparted by Apa was comparable to that induced by Ag85A. Single live BCG vaccination by either route also imparted significant protection compared to naïve mice, however, protection imparted by i.n. and s.c BCG vaccination did not differ significantly from each other. The results comparing routes of BCG vaccine were similar in three independent experiments (data not shown). There was also no significant difference in the number of mycobacterial colonies on plates with or without 2-thiophene carboxylic acid hydrazide (TCH), indicating no detectable residual BCG in the lungs or spleens 6 weeks after challenge (data not shown). Subunit vaccination imparted comparable protection to BCG when mice were challenged 8 weeks after first subunit dose (Fig. 5A). However, significantly better protection (Student's *t* test and Mann-Whitney *U* test p<0.05) was observed with BCG vaccination (i.n. or s.c.) as compared to Apa and Ag85A-based subunit vaccines when mice were challenged 12 weeks after first subunit vaccine dose (Fig. 5B).

### Intranasal Apa vaccine induces strong in vitro T-cell recall responses

**Figure 6:**
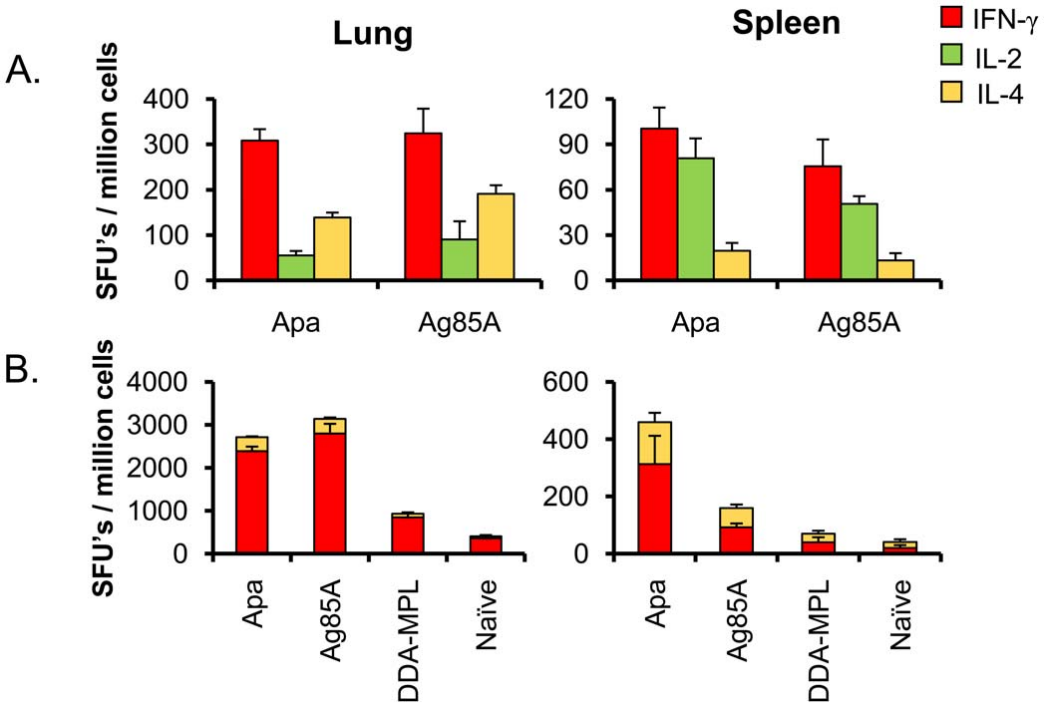
Recall responses induced by Apa subunit vaccination. BALB/c mice were vaccinated with three different doses of Apa in DDA-MPL adjuvant at two week intervals and compared with Ag85A/DDA-MPL vaccinated and naïve (unvaccinated) and sham (adjuvant only) vaccinated control mice. Eight weeks after first dose (or four weeks after last dose), before *M. tuberculosis* challenge, mice were sacrificed and immunogen-specific T-cell responses were investigated *in vitro* in the lungs and spleen by ELISPOT assay. The frequencies IFN-γ, IL-2 and IL-4 cytokine-secreting cells are expressed as spot forming units (SFUs)/million cells of organ after *in vitro* restimulation of 1×10^5^ cells/well from each organ with either **A.** respective immunogen pulsed or **B.** *M. bovis* BCG Copenhagen infected BM-DCs (multiplicity of infection 1∶1) at the ratio of 5∶1 organ cells/DC for 40 h. The results are calculated as means ± standard deviation of triplicate determinations of pooled cells from four mice after subtracting the SFUs from respective unstimulated cultures.

Use in multicomponent vaccine formulations might result in both potentiating and suppressive interactions between the individual components, or competition between various components of complex vaccines (such as live BCG or multicomponent subunit vaccine) for processing and presentation can lead to either increased or decreased immunogenicity of any component [41]. Therefore, we investigated the magnitude of recall responses induced by Apa subunit vaccine when administered alone, and compared with that induced by Ag85A subunit vaccine. Evaluation of immunogen-specific *in vitro* recall response at 4 weeks following the last dose (the time interval used for *M. tuberculosis* challenge in the protection studies) demonstrated that both Apa and Ag85A vaccination induce comparable levels of IFN-γ, IL-2 and IL-4 responses in the lungs and spleen (Fig. 6A), and in the CLN (data not shown), as evaluated by ELISPOT assay. These results suggest that Apa vaccination induces a similar magnitude of T-cell recall responses as Ag85A-based subunit vaccine before challenge. When the lung cells or splenocytes of vaccinated mice were incubated with bone marrow-derived dendritic cells that had been pulsed with *M. bovis* BCG *in vitro* (as a surrogate for *M. tuberculosis* challenge) a strong IFN-γ recall response was elicited in lung as compared to spleen and was characterized by increased IFN-γ SFU's compared to IL-4 (Fig. 6B). These results suggest the ability of i.n. Apa and Ag85A subunit vaccines to induce strong IFN-γ recall immune responses in the lungs after *M. tuberculosis* challenge.

## Discussion

Our study demonstrate that it is possible to engender a robust mycobacterium-specific immune response in the lungs by i.n. instillation of a single moderate dose of BCG vaccine, including strong type 1 (IFN-γ and IL-2) lymphocyte responses and macrophage activation (as measured by increased NO release). Our results also demonstrate that i.n. BCG vaccination induces specific T-cell responses in the CLN, which drains the NALT and nasal cavity, while s.c. delivery produces responses almost exclusively in the draining ILN. These observations corroborate the tenet that the immune system is compartmentalized and the early immunological responses tend to be strongest in compartments most proximal to the site of vaccine administration.

Interestingly, the frequency of antigen-specific cytokine-secreting cells found in the lymph nodes (CLNs and ILNs), a secondary lymphoid organ where immune responses develop, was low compared to that found in a nonlymphoid tissue such as the lung. This may be explained by the propensity of effector and memory T-cells to migrate out of LNs and preferentially reside in nonlymphoid organs [42]. In our study, long lasting T cell immunity was found to be augmented not only in both inductive (NALT and CLN) and effector sites (lung and spleen) but also in an unrelated cavity (peritoneal cavity) and migratory memory T-cell nesting compartment (BM) [43]. The cytokine responses were also observed at the level of MLN that drains the gastrointestinal tract (another mucosal surface), collectively suggesting that i.n. BCG is capable of inducing immunity in multiple local and systemic immune compartments.

The ELISPOT results further demonstrated the ability of i.n. BCG vaccination to induce strong and sustained mycobacterium-specific immunity, directed toward both secreted and subcellular fractions of *M. tuberculosis*. These observations, in part, were in agreement with ELISPOT results observed by Goonetilleke *et. al* [23] using PPD as an *in vitro* stimulus where peak IFN-γ responses were observed at 12 weeks after parenteral and i.n. BCG vaccination and were sustained until 24 weeks. However, our observations differ from those of Chen *et. al.* [14] who demonstrated peak IFN-γ levels at 3 weeks post i.n. BCG vaccination that declined rapidly after 6 weeks as measured by cytokine ELISA using killed BCG as *in vitro* stimulus.

It has been previously shown that early BCG multiplication occurs in the draining ILN following s.c. vaccination at the base of tail [44]. BCG then disseminates in the spleen and lungs after 4 weeks with significantly more bacilli counts in the spleen than in the lungs [44]. On the contrary, large portion of the total BCG delivered could be cultured from the lungs within 24 h of i.n. BCG vaccination [23], [45]. This results in more BCG load in the lungs than in the spleen of mice [45]. Although we have not studied BCG persistence and dissemination in different local and distant organs in detail after i.n. or s.c administration, we observed similar BCG growth kinetics in the lungs and spleen as reported in above studies over a course of 12 weeks depending on the route of vaccination (data not shown). These differences in the BCG bacillary antigenic load in the draining lymph nodes, lungs and spleen after i.n. and s.c vaccination may account for the differences observed in the magnitude of specific immune responses in these organs.

Despite observed differences in the frequencies and location of specific cytokine secreting T cells both BCG vaccination routes afforded comparable levels of protection against airway *M. tuberculosis* challenge in our study. It can be inferred that the frequency of T cells in the lungs elicited by s.c. BCG vaccination was sufficient to afford protection equivalent to that of i.n. BCG in the lungs. Increased ratio of specific IFN-γ to IL-4 SFU's in the lungs after i.n. BCG vaccination also did not contribute toward the enhancement of protection compared to s.c. BCG. These results suggest that the protection imparted by a single moderate dose of BCG vaccine is independent of route of vaccination in our model. It also indicates that the readout of correlate of protection against TB may not be as simple as induction of dominant type 1 cytokine response.

Our results are contrary to the findings of earlier studies in which i.n. BCG afforded better protection than s.c. vaccination [14], [46]. However, these results in part correlate with the findings of Goonetilleke *et. al.* [23], who demonstrated that a single i.n. dose of BCG did not differ in the degree of protection afforded and that two sequential i.n. BCG doses are required to afford superior protection compared to parenteral BCG vaccine administered using similar double dosing strategy. In different studies, intravenous, oral, rectal and aerosol BCG administration using single dose imparted similar protection against *M. tuberculosis* [47], [48], [49]. It has been shown that BCG vaccination using wide range of dose induces varying magnitude and quality of immune responses but imparts similar levels of protection [50], [51]. Thus, the route of application and consequently the dissemination and tissue localization of BCG following a single vaccination may have minor impact on protective immunity against TB [49].

However, similar conclusion may not be drawn for non-multiplying subunit or killed vaccines and detailed studies evaluating effect of route, dose and delivery system on the protection imparted by individual subunit vaccines are required. Mice vaccinated via i.n. route using culture filtrate proteins-based subunit vaccine were found to be better protected as compared to s.c. route [52], and the dose of a subunit vaccine formulation has been shown to critically influence its immunogenicity and protective efficacy [53]. Furthermore, one cannot totally discount the benefit of mucosal immunity induced by a single i.n. BCG as the compartmentalization of immune responses generated in the lungs and respiratory tract has been shown to have positive implications for the development of booster subunit vaccines and effective prime-boost vaccination strategies [23], [25].

Central to the development of a subunit vaccine would be identification of *M. tuberculosis* proteins that can effectively prime mycobacterium-specific immunity in the respiratory tract. Increased immune responses in the lungs and CLN after i.n. vaccination enabled us to identify *M. tuberculosis* proteins recognized by the immune cells of these organs. Among the nine proteins evaluated in this study, Apa and GroEL were found to be highly antigenic in BCG-vaccinated mice as measured by IFN-γ response in different immune compartments. Other polypeptides which induced prominent responses following i.n. BCG vaccination were Ag 85 complex proteins and Pst-S1. The magnitude and hierarchy of these antigen-specific responses varied at different time points, which may be due to stage specific differential expression of antigens *in vivo* or variation in subcellular localization of bacilli resulting in the temporal differences in the antigen profile recognized. Of note, Apa, a 45/47 kDa cell surface or secreted glycoprotein, GroEL, a 65 kDa cytosolic heat shock protein and Ag85 complex, 30–32 kDa cell surface or secreted polypeptides, have all been previously shown to be highly antigenic in humans [35], [54], [55] with different stages of *M. tuberculosis* infection.

The Apa complex is composed of mannosylated proteins [56] with up to nine identified glycoforms [57], [58]. APA is also known as *M. tuberculosis* protein (MPT)-32 (calculated molecular mass 32 kDa), ModD (putative involvement in molybdate uptake) or fibronectin attachment protein (FAP), and can mediate bacterial attachment to host cells as a potential adhesin [59]. Both Apa and Ag85 complex proteins have been found to be released into phagosomes and other subcellular compartments of *M. bovis* BCG infected macrophages [60]. The recombinant Apa homolog has also been found to activate dendritic cells and induce Th1 polarization [61]. It is noteworthy that Apa is predominantly recognized following vaccination with live but not dead BCG organisms [62]. Moreover, monoclonal antibody against Apa has been previously shown to abrogate the attachment and internalization of BCG by human bladder tumor cells and stable binding of BCG to bladder mucosa via FAP was necessary for the expression of BCG-induced antitumor activity [63], [64]. Overall, this suggests that APA-specific responses might contribute to protective responses induced by live multiplying BCG.

In parallel with the responses following i.n. BCG vaccination, Apa was also highly immunogenic after intranasal multicomponent subunit vaccination. The immune responses induced by Apa were characterized by strong *in vitro* type1 and type 2 cytokine response. The Apa also induced strong antibody response characterized by elevated specific serum IgG and nasal lavage IgA as determined using ELISA and cytotoxic T-cell response (mean cytotoxicity 30%) as evaluated by neutral red uptake assay (data not shown).

It has been previously shown that deglycosylation of Apa affects its capacity to stimulate *in vitro* proliferation of T-cells from guinea pigs following s.c. BCG vaccination [58]. However, i.n. recombinant (unglycosylated) Apa subunit vaccinated mice could significantly inhibit *M. tuberculosis* growth in the lungs and spleen similar to that of Ag85A-based subunit vaccine (this study) and a s.c. prime-boost vaccination strategy using recombinant MVA expressing Apa has been previously shown to protect guinea pigs against virulent *M. tuberculosis* challenge [35]. These observations along with the strong antigenicity and immunogenicity obtained from the recombinant (unglycosylated) Apa used in this study warrants further evaluation in comparison with its native (glycosylated) counterpart.

The significant level of protection afforded in animal models by Ag85A, another fibronectin binding protein, after i.n. vaccination in previous studies [23], [24] and strong T cell mediated immunogenicity elicited by Apa vaccination in the respiratory and other immune compartments leading to comparable protection against *M. tuberculosis* challenge in the current study bodes well for use of these molecules as components of future mucosal TB vaccines. Overall, findings of this study strongly support further evaluation of mucosally targeted Apa-based vaccine to prevent tuberculosis.

## Materials and Methods

### Animals

Specific-pathogen-free, 6–8 weeks old female BALB/c (H-2^d^) mice purchased from Harlan Sprague Dawley (Indianapolis, IN) were used in the study. All mice were housed in Biosafety Level-II or -III animal facility, as per the requirement of the experiment. All animals were fed on a standard pellet diet and water *ad libitum*. Experiments performed were in accordance with the approved animal protocols (permit numbers: 1422SHINMOUC, 1664SABMOUC, 1490SABMOUC and 1847SABMOUC) and guidelines of the Institutional Animal Care and Use Committee (IACUC) of CDC, Atlanta.

### BCG vaccine and *M. tuberculosis* culture

The BCG (Copenhagen) vaccine and *M. tuberculosis* Erdman were provided as a TB preclinical vaccine reference standard and challenge strain, respectively by the Center for Biologics Evaluation and Research, Food and Drug Administration (FDA), Bethesda, USA.

### *M. tuberculosis* antigens

Nine recombinant *M. tuberculosis* H37Rv proteins (Table 1), whole cell lysate (WCL), and total short term culture filtrate proteins (STCF) were obtained through the TB Vaccine Testing and Research Material (TBVTRM) contract funded by National Institute of Health (NIH)/National Institute of Allergy and Infectious Diseases (NIAID) at Department of Microbiology, Immunology and Pathology, Colorado State University, Fort Collins, Colorado. Nine recombinant plasmids containing *M. tuberculosis* genes that encode the nine proteins (Table 1) were also obtained. Some of the recombinant proteins were further expressed in *E. coli* BL-21 (DE3) and purified as per the protocol provided by the TBVTRM contract.

### BCG and subunit vaccination

Lyophilized BCG vaccine was resuspended in vaccine diluent (diluted Sauton medium) provided by the supplier (Statens Serum Institute, Copenhagen, Denmark). For s.c. vaccination, 50 µl of a BCG suspension (7×10^5^ CFUs) was injected above the gluteus superficialis and biceps femoralis muscles of both hind legs using a 26 gauge needle. Intranasal administration was carried out by applying a total of 30 µl of BCG vaccine (7×10^5^ CFUs) to the external nares (15 µl per nostril) using a fine tip micropipette and allowing the mouse to inhale the suspension into the lungs naturally. BCG vaccine diluent was used as a control for either route of vaccination.

For multicomponent subunit vaccination, mice were immunized by the i.n. route three times at 2-week intervals as described above except using 90 µg of recombinant *M. tuberculosis* protein mixture per dose [10 ug of each immunogen emulsified in DDA (250 µg/dose, Sigma-Aldrich, St. Louis, MO) and MPL (derived from *Salmonella minnesota* Re 595; 25 µg/dose, Sigma-Aldrich)]. The emulsion was prepared as described previously [28]. Briefly, MPL was first mixed with endotoxin-free sterile water (Burdick & Jackson, Muskegon, MI) containing 0.2% triethylamine (Fisher Scientific, Fair Lawn, NJ). The mixture was heated in a 70°C water bath for 30 s and then sonicated for 30 s. The heating and sonicating procedure was repeated twice. To prepare the emulsion, DDA was suspended in sterile water and a homogeneous dispersion of the powder was obtained by heating the suspension at 80°C for 5–10 min in water bath. After cooling to room temperature, MPL and antigens were mixed with DDA just before use. The sham-immunized mice received PBS (pH 7.2) emulsified in DDA-MPL.

### Collecting tissues

Following vaccination, mice were bled by cardiac puncture under anesthesia and sacrificed at different time points. Lungs, spleen, NALT, CLN (superficial), ILN, MLN and femur and tibial bone marrow (BM) were aseptically removed and placed into RPMI 1640 supplemented with 100 IU ml^−1^ penicillin, 50 µg ml^−1^ streptomycin, 1 mM l-glutamine, 25 mM HEPES, 1 mM sodium pyruvate, 5×10^−5^ M ß-mercaptoethanol, vitamins and nonessential amino acids (Gibco-Invitrogen, Grand Island, NY) and 10% endotoxin-tested heat-inactivated fetal calf serum (FCS; Atlas Biologicals, Fort Collins, CO). Thoracic and peritoneal exudate cells were isolated by washing the respective cavities with RPMI 1640 media. In each case, the respective lymphoid or extra-lymphoid organs or cavity washings were collected from 4 mice for each treatment group and cells were extracted for analysis of *in vitro M. tuberculosis* antigen-specific cellular responses.

### Isolation of immune cells

To isolate lung cells, mice were bled by cardiac puncture under anesthesia and their lungs were perfused via the right ventricle with PBS containing 10 U ml^−1^ heparin to remove intravascular leukocytes. The lungs were then perfused with an enzyme mixture containing 1 mg/ml collagenase type IV (Sigma-Aldrich) and 25 U ml^−1^ DNase (Roche, Penzberg, Germany) in supplemented RPMI and sliced into small pieces in a sterile dish and the fragments were incubated in the enzyme mixture at 37°C for 1 h. The digested lung fragments were pressed with a 5 ml syringe plunger through a 70-µm pore size cell strainer (BD Falcon, Bedford, MA) to obtain a single cell suspension and the erythrocytes were lysed with RBC lysis buffer (eBioscience, San Diego, CA) for 4–5 min at room temperature. The lung cells were washed, recovered by centrifugation, and resuspended in supplemented RPMI for counting using an automated cell counter (Countess, Invitrogen, Carlsbad, CA) employing the trypan blue dye exclusion method. The NALT was isolated as described previously by Asanuma *et. al.* [65] and BM was isolated by flushing cavities of femurs and tibias with RPMI. The single cell suspensions of spleen, lymph nodes, BM or NALT were obtained by gently grinding the respective organs through a 70-µm cell strainer into 10–20 ml supplemented RPMI. The cell suspensions were centrifuged at 300×g for 10 min and the erythrocytes were removed by treatment with RBC lysis buffer when necessary. Cells were washed several times with fresh RPMI and the cell concentration was adjusted accordingly.

### BM derived dendritic cells (DCs)

These were generated from naïve BALB/c BM according to the procedure described by Inaba *et. al.* [66]. A granulocyte-macrophage colony stimulating factor (GM-CSF) expressing J558L cell line (supplied by Dr I. Mellman, Yale University School of Medicine, New Haven, CT) was used as a source of GM-CSF. Supplemented RPMI containing recombinant interleukin (IL)-4 (20 ng ml^−1^, PreproTech, Rocky Hill, NJ) and GM-CSF (1∶200 dilutions) was used to culture BM DCs. Flow cytometric analysis revealed that >90% of cells were CD11c^+^ after 7 days of culture.

### Enzyme linked immuno-spot (ELISPOT) assay

Commercially available interferon (IFN)-γ, IL-2, and IL-4 kits (Mouse ELISPOT set; BD-Biosciences, San Diego, CA) were used to enumerate frequencies of *M. tuberculosis* antigen-specific cells according to the manufacturer's protocol. In brief, 96 well ELISPOT plates were coated with 100 µl of 5 µg ml^−1^ capture antibody in PBS (pH 7.2) and incubated overnight at 4°C. Free binding sites were blocked with 200 µl of supplemented RPMI containing 10% FCS for 2 h at room temperature. Cell concentration was adjusted to 0.5–2×10^6^ cells ml^−1^ for all sites except 0.5×10^6^ cells ml^−1^ for NALT and added to appropriate wells. BM-derived DCs were added at the ratio of 5∶1 immune cells/DC to supplement the antigen presenting cells already present in the cell suspension. For each treatment group, cells were stimulated in triplicate with either 10 µg ml^−1^ of individual recombinant purified *M. tuberculosis* antigens, antigen combination, STCF, WCL, Concanavalin A (Con-A; Sigma-Aldrich), Tetanus toxoid (TT; Calbiochem-EMD Biosciences, San Diego, CA), Isopentenyl pyrophosphate (IPP; Sigma-Aldrich) or medium alone in a 100 µl volume. After 36–40 h of incubation at 37°C in a humidified atmosphere containing 5% CO_2_, the unattached cells were aspirated from the well and the remaining cells were lysed with distilled water. The wells were washed again with PBS containing 0.05% Tween-20 (PBS-T) and the site of cytokine secretion was detected with a biotin-labeled anti-mouse cytokine antibody and horseradish peroxidase-conjugated streptavidin. The enzyme reaction was developed using 3-amino-9-ethylcarbazole (AEC) substrate reagent set (BD-Bioscience). The number of spot forming units (SFUs) per well were counted automatically using an ELISPOT reader (Cellular Technology Limited, Cleveland, OH). The number of spots specific for each antigen preparation was calculated by subtracting the number of spots that formed in the absence of added antigen from the number that formed in its presence.

### Lymphocyte proliferation assay

Cells isolated from different sites were seeded in sterile 96-well flat-bottom tissue culture plates (Costar, Corning, NY) at 1×10^6^ cells ml^−1^ in 100 µl of supplemented RPMI-1640. BM derived DCs were used at the ratio of 5∶1 lymphocytes/DC as described for ELISPOT assay in appropriate experiments. For each treatment group, cells were stimulated in triplicate with either 100 µl of 10 µg ml^−1^ of purified recombinant *M. tuberculosis* antigens, antigen combination, WCL, STCF, or Con-A in supplemented RPMI as a positive control for cell viability and reactivity or medium alone as a negative control. Cultures were incubated in a humid atmosphere containing 5% CO_2_ at 37°C for 72 h. 1 µCi of ^3^H-thymidine (Perkin Elmer, Wellesley, MA) was added to each well and after 18–20 h incubation the cells were harvested on glass fiber filters (Perkin Elmer) using an automated cell harvester (TOMTEC, Inc. Hamden, CT). Once dry, the radioactivity incorporated was counted using a β-scintillation counter (Perkin Elmer). The proliferation was expressed as mean counts per minutes (CPM) of antigen stimulated cultures after subtracting mean counts per minutes of cultures without antigen and the stimulation index (SI) was calculated by dividing mean counts per minute in antigen-stimulated wells by mean counts per minute in unstimulated wells.

### Determination of nitrite accumulation

Nitrite (NO_2_^−^) accumulation in the supernatant of cultured cells was measured as an indicator of nitric oxide (NO) production by a Griess assay using a sodium nitrite standard as described previously [55]. Supernatants (100 µl) from 1×10^6^ cells ml^−1^ of each condition stimulated with 10 µg ml^−1^ WCL, *E. coli* LPS-TLR-4 ligand (InvivoGen, San Diago, CA) or RPMI medium alone after 96 h of culture at 37°C were assayed in triplicate, and the absorbance was measured at 550 nm.

### Multiplexed microsphere-based cytokine immunoassay

The supernatants of cell cultures stimulated with *M. tuberculosis* antigens were harvested after 72 h of culture, centrifuged, filtered, and stored at −70°C, and used in a multiplexed microsphere-based cytokine immunoassay. Duplicate cell culture samples were assayed in duplicate using a Bio-Plex Cytokine Assay kit (Bio-Rad Laboratories; Hercules, CA) as per the manufacturer's instructions. The median fluorescence intensity (MFI) was determined on a Bio-Plex 100 instrument equipped with Bio-Plex Manager v. 4.0 software. The concentrations for IL-2, IL-4, IL-5, IL-10, IL-12 p70, TNF-*α* and GM-CSF are reported as pg/ml.

#### Experimental infections

Protective efficacy was evaluated in two separate challenge experiments. In both cases, mice were challenged by the intranasal inhalation route to deliver 4×10^4^ CFUs of *M. tuberculosis* Erdman by instilling a total of 30 µl in to the external nares (15 µl per nostril). At 48 h following challenge, approximately 0.5% of the total CFUs delivered could be cultured from the lungs. In the first experiment, mice were immunized intranasally with three doses of recombinant Apa or Ag85A (10 µg/dose) in DDA-MPL adjuvant at 2-week intervals. The immunogen-specific recall immunity was investigated 4 weeks after the last dose *in vitro* in a separate experiment using similar immunization protocol. In the second experiment, mice received three doses of subunit vaccine at 4-week intervals. In both cases, mice were challenged 4 weeks after the last immunization and CFU levels were evaluated at week 6 post-infection. Bacterial levels were determined by plating serial dilutions of lung and spleen homogenates onto Middlebrook 7H10 agar (BD-Biosciences) supplemented with OADC and TCH (2 µg ml^−1^). Colonies were enumerated after 4 weeks of incubation at 37°C and numbers were expressed as the log_10_ values of the geometric mean for five mice. Protective efficacy promoted by the Apa/DDA-MPL and Ag85A/DDA-MPL subunit vaccines was compared with the protective levels afforded by the conventional TB vaccine, BCG. To this end, mice received one dose (7×10^5^ CFUs) of BCG injected subcutaneously on the hind legs or instilled intranasally (as described above) at the time of first dose of subunit vaccines and protective efficacy was analyzed in parallel with the subunit vaccinated groups.

### Statistical analysis

The data obtained from immunological assays were analyzed using a Student's *t*-test and analysis of variance (ANOVA). Differences between the different groups in the protection experiments were assessed by one-way ANOVA of the log_10_ CFUs followed by Tukey's test and also by its nonparametric equivalent Kruskal-Wallis test (for comparison of more than two groups) and Mann-Whitney *U* test (for comparison of two groups). A value of p<0.05 was considered significant.

## Supporting Information

Figure S1 **T-cell responses in the CLN of intranasally BCG-vaccinated mice.** The frequencies of nine antigen-specific Th1 (IFN-γ and IL-2) and Th2 (IL-4) cytokine secreting cells in the CLN of i.n. BCG vaccinated mice at early (3 weeks) and late (30 weeks) time points were enumerated by ELISPOT assay and expressed as SFUs/million cells. The assay was developed after stimulation of 1×10^5^ cells/well for 36–40 h with individual antigens in the presence of BM-DCs at the ratio of 5∶1 lung cells/DC. The results are calculated as means ± standard deviation of duplicate determinations of pooled cells from four mice after subtracting the SFUs from respective unstimulated cultures. Data presented are representative of two separate experiments each consisting of four mice per group.
(TIF)

Figure S2 **T-cell responses in the CLN, PC and MLN of intranasally multicomponent subunit-vaccinated mice.** The frequencies of immunogen-specific Th1 (IFN-γ and IL-2) and Th2 (IL-4) cytokine-secreting cells in the CLN, PC and MLN two weeks after intranasal multicomponent vaccination as enumerated by ELISPOT assay using BM-DCs as antigen presenting cells in the cell culture and expressed as SFUs/million cells of organ. The results are presented as means ± standard deviation of four determinations of pooled cells from four mice after subtracting the SFUs from respective unstimulated cultures.
(TIF)
