# Supplementary figures and images for: Cellular Immune Responses to Nine Mycobacterium tuberculosis Vaccine Candidates following Intranasal Vaccination
Source: PLoS One. 2011 Jul 25;6(7):e22718. doi: 10.1371/journal.pone.0022718 (PMC3143185; doi:10.1371/journal.pone.0022718)

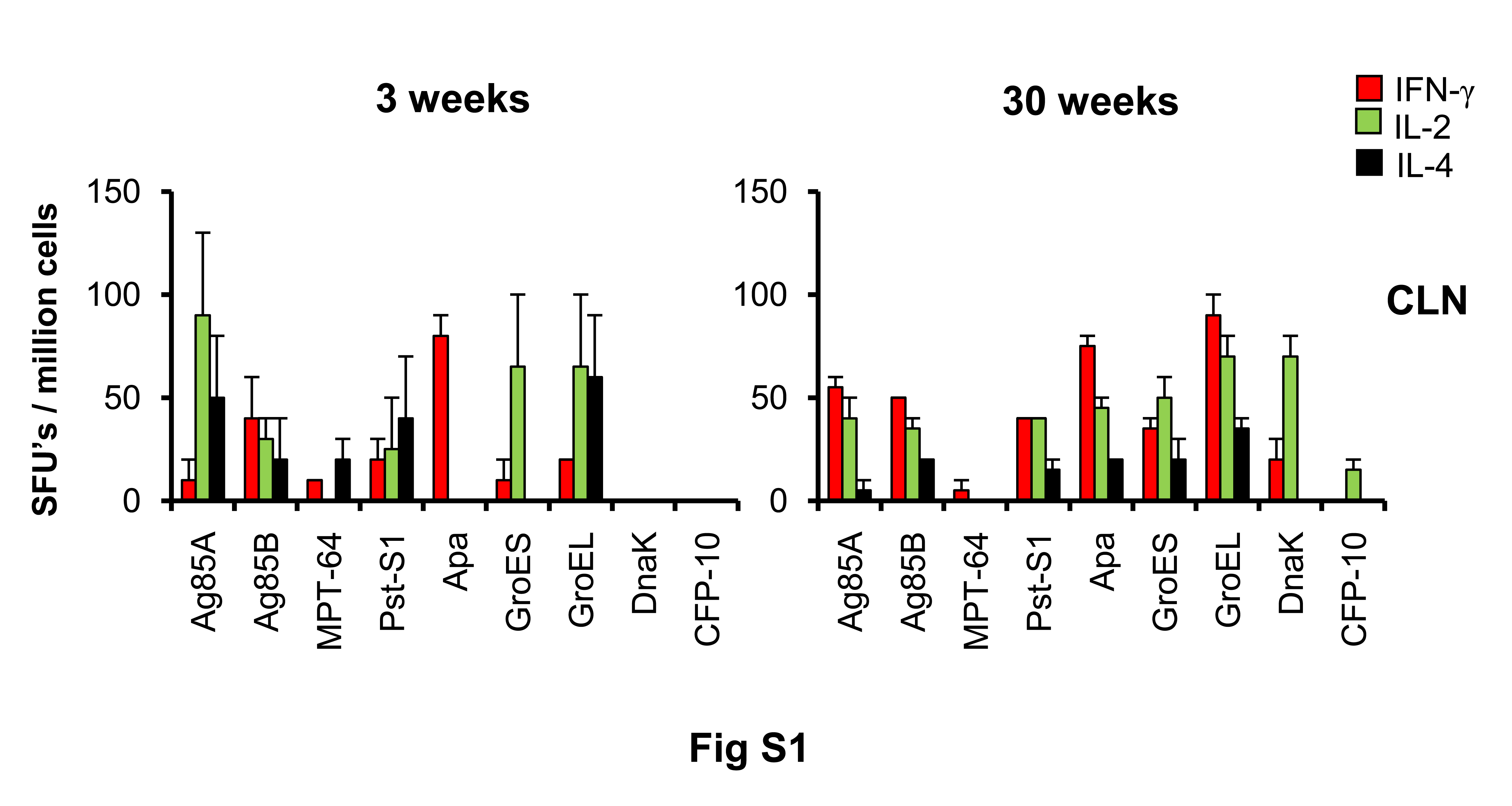

Supplement: Figure S1 — T-cell responses in the CLN of intranasally BCG-vaccinated mice. The frequencies of nine antigen-specific Th1 (IFN-γ and IL-2) and Th2 (IL-4) cytokine secreting cells in the CLN of i.n. BCG vaccinated mice at early (3 weeks) and late (30 weeks) time points were enumerated by ELISPOT assay and expressed as SFUs/million cells. The assay was developed after stimulation of 1×10^5^ cells/well for 36–40 h with individual antigens in the presence of BM-DCs at the ratio of 5∶1 lung cells/DC. The results are calculated as means ± standard deviation of duplicate determinations of pooled cells from four mice after subtracting the SFUs from respective unstimulated cultures. Data presented are representative of two separate experiments each consisting of four mice per group. (TIF) [file pone.0022718.s001.tif]

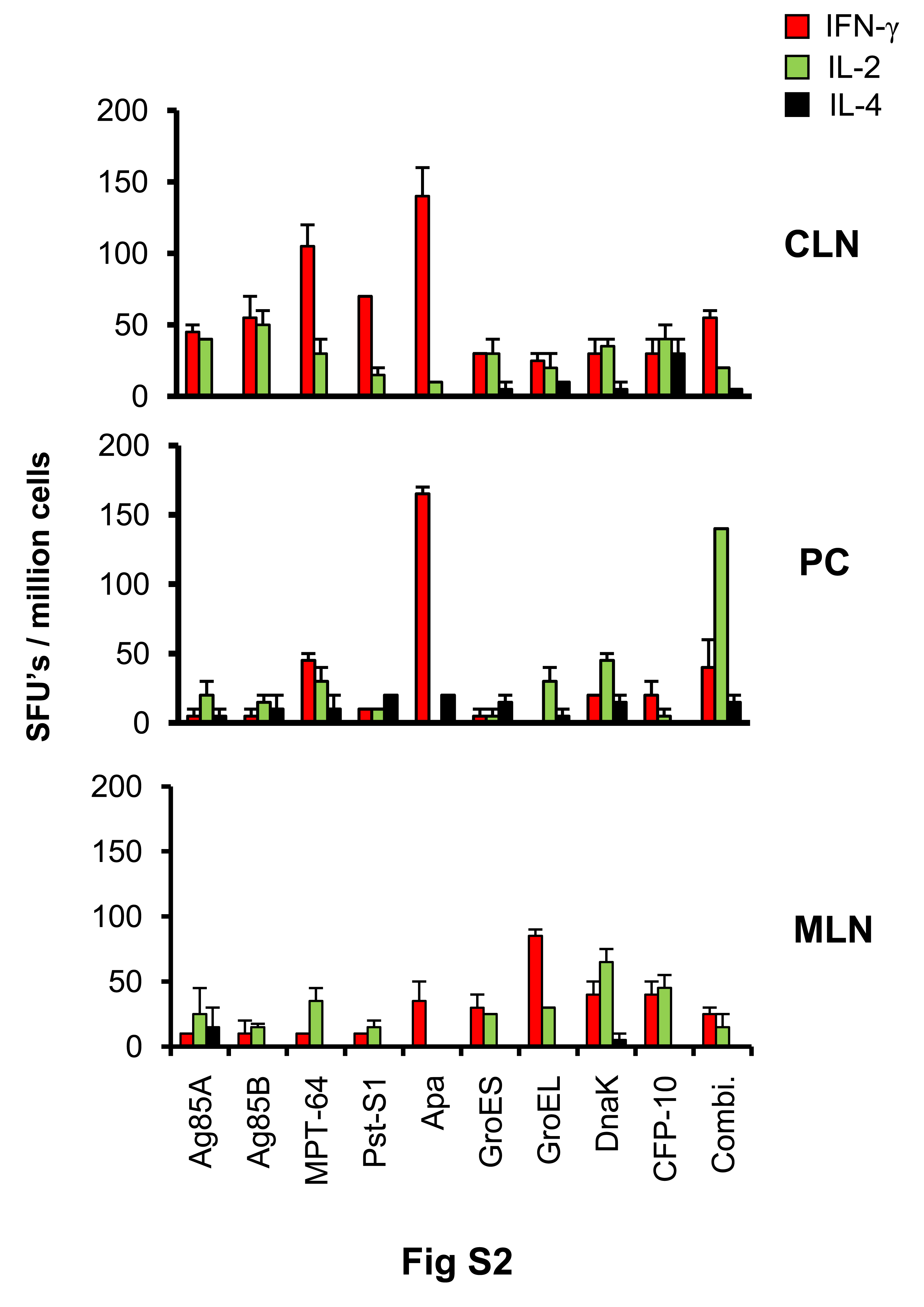

Supplement: Figure S2 — T-cell responses in the CLN, PC and MLN of intranasally multicomponent subunit-vaccinated mice. The frequencies of immunogen-specific Th1 (IFN-γ and IL-2) and Th2 (IL-4) cytokine-secreting cells in the CLN, PC and MLN two weeks after intranasal multicomponent vaccination as enumerated by ELISPOT assay using BM-DCs as antigen presenting cells in the cell culture and expressed as SFUs/million cells of organ. The results are presented as means ± standard deviation of four determinations of pooled cells from four mice after subtracting the SFUs from respective unstimulated cultures. (TIF) [file pone.0022718.s002.tif]
